# 

**Published:** 1963-10

**Authors:** 


### *Contents*

| | | |
|---|---|---|
| MEDICINE IN NORTH AMERICA | *J. Apley* | 109 |
| HAEMATURIA AND OFFICE UROLOGY | *H. Guerrier* | 114 |
| A FOETUS WITH DOWN'S SYNDROME | *M. B. Wingate* | 121 |
| LAST CONSCIOUS THOUGHTS | *G. De M. Rudolf* | 125 |
| CLASSIFICATION OF JUVENILE DELINQUENTS | *F. Bodman* | 127 |
| OBITUARY | | 131 |
| SOUTH WEST SURGEONS | | 132 |
| WESSEX RAHERE CLUB | | 132 |
| EXAMINATION RESULTS | | 133 |
| CORRESPONDENCE | | 134 |

